# Requirements and key factors for the successful implementation of AI in clinical practice: a qualitative study from the European CAIDX network

**DOI:** 10.3389/fdgth.2026.1954586

**Published:** 2026-10-27

**Authors:** Doreen Goerss, Carina Østervig Byskov, Stina B. Jonasson, Ida Lyngholm, Krzysztof Tupikowski, Surenth Nalliah, Stefan Jovinge, Jaanus Pikani, Stefan Teipel, Martin Dyrba

**Affiliations:** 1 Department of Psychosomatic Medicine, Rostock University Medical Center, Rostock, Germany; 2 German Center for Neurodegenerative Diseases (DZNE), Rostock, Germany; 3 The Innovation Clinic, Aalborg University Hospital, Aalborg, Denmark; 4 Department of Research, Development, Education and Innovation, Skåne University Hospital, Lund, Sweden; 5 AUH Innovation & International Affairs, Aarhus University Hospital, Aarhus, Denmark; 6 Subdivision of Urology, Lower Silesian Oncology Pulmonology and Hematology Center, Wroclaw, Poland; 7 Department of Oncology and Hematology, Faculty of Medicine, Wrocław University of Science and Technology, Wroclaw, Poland; 8 Department of Radiology, Aalborg University Hospital, Aalborg, Denmark; 9 Department of Clinical Medicine, Aalborg University, Aalborg, Denmark; 10 Cardiovascular Institute, Stanford University, Palo Alto, California, CA, United States; 11 Tartu Biotechnology Park, Tartu, Estonia

**Keywords:** artificial intelligence, Health Technology Assessment, healthcare, implementation research, qualitative research

## Abstract

**Introduction:**

Artificial intelligence (AI) is expected to become increasingly important in hospitals and clinical care. However, successful implementation depends on technical, organizational, and user-related factors. We aimed to identify the key requirements for equitable and effective adoption of AI-based applications in clinical practice.

**Methods:**

Within the “*Clinical AI-based Diagnostics”* (CAIDX) project, we conducted systematic expert interviews with 55 stakeholders from four European countries working across the healthcare sector. Interviews explored the perceived added value of AI, barriers to implementation, usability requirements, and potential health-economic implications.

**Results:**

Stakeholder analysis revealed several recurrent sources of tension affecting implementation: limited physician involvement, differing perspectives between IT specialists and clinicians, unclear distribution of tasks and responsibilities, and low institutional commitment driven by concerns about inefficient use of time and resources. We also identified structural and workforce-related constraints that make implementation particularly challenging, including the large number of actors involved and the highly individual organizational setup of hospitals, which limits transferability of implementation models.

**Discussion:**

Implementation of clinical AI requires targeted action at hospital, vendor, and health-system levels. Compared with earlier healthcare technologies, AI adoption is shaped more strongly by AI literacy, professional attitudes, and perceptions of trust and responsibility. Based on these findings, we propose practical recommendations to support implementation of AI applications in routine care.

## 1 Introduction

Over the past decade, the development and availability of medical artificial intelligence (AI) applications have grown rapidly (1–3). Currently, there are numerous novel algorithms with promising applications in radiology, pathology, oncology and intensive care medicine (4–6). Despite this rapid progress, only a small proportion of European hospitals routinely use AI-based applications in clinical practice (6, 7). Surveys indicate that the implementation rate still lags far behind both the potential and the number of available systems (8). One reason for this is that, although many AI systems have been proposed, only a small number of independent clinical validation studies have provided evidence regarding their technical accuracy, sensitivity and specificity. See (9) for an extensive review and https://healthairegister.com/ for a continuously updated register.

Beyond technical accuracy, the effectiveness of AI in clinical settings should be understood as a multidimensional concept that includes clinical benefit, patient safety, robustness across populations and care environments, usability, integration into clinical workflows, transparency, and appropriate human oversight (10). These dimensions are particularly relevant because AI systems may generate outputs resembling autonomous diagnostic judgments, raising concerns about trust, liability, misuse, and accountability when clinicians and AI assessments differ (11, 12). The successful translation of AI from research or industry into routine hospital practice therefore depends not only on model performance but also on traceability and explainability, reliable data governance, clearly defined responsibilities, and evidence that the system improves rather than complicates clinical decision-making (10, 12, 13). This perspective aligns with the EU AI Act's risk-based approach to high-risk AI systems used in healthcare (14), as well as WHO guidance emphasizing safety, effectiveness, transparency, equity, accountability, and sustained human control throughout the AI lifecycle (15).

For instance, recent evidence for AI-based breast cancer detection in mammography shows an overall increase in the cancer detection rate (16, 17). However, this comes at the cost of a higher false-positive rate. Although the time a radiologist spends evaluating an individual mammogram decreases, their overall workload increases due to the additional time required to check the false-positives. Therefore (17), emphasized the importance of adaptive calibration and continuous monitoring of such AI systems to ensure overall efficiency, safety, and equity.

Economic barriers are also important to address. Many physicians or hospitals operate under significant financial pressure, so they only adopt costly AI solutions if clear clinical and/or economic benefits are demonstrated (8). At the same time, experience shows that AI applications with strong performance in controlled settings often fail to achieve comparable effectiveness in routine practice due to various reasons such as varying data quality or differences in procedures and workflows (13).

The data for this study was acquired as part of the ‘Clinical AI-Based Diagnostics’ (CAIDX) project. CAIDX aims to help healthcare professionals integrate artificial intelligence (AI) into clinical practice by supporting the development and clinical implementation of AI, thereby improving diagnostics and treatment. The CAIDX project was funded by Interreg Baltic Sea Region program of the European Union. The consortium consists of thirteen partners, including five hospitals and eight other organizations, such as business support organizations, higher education and research institutions, from six EU countries: Denmark, Poland, Germany, Estonia, Finland and Sweden. More information about the CAIDX project can be found online at: https://clinicalai.eu.

This study investigated how successful translation of medical AI applications can be achieved in European healthcare. This required a systematic analysis of barriers in the implementation process and the identification of best practice approaches for all stakeholders, including clinical staff, the hospital management, providers, legal and IT departments, and regulatory institutions (14). We present a comprehensive and practical analysis of the pivotal factors that contribute to the successful integration and utilization of medical AI applications within the European healthcare context.

## 2 Materials and methods

### 2.1 Overview and interviewees

The data collection encompassed four steps. First, the prior experiences of AI-related implementation projects were systematically collected among the CAIDX hospital partners. Based on these experiences, research questions were formulated to design the guidelines for semi-structured expert interviews. Then, in expert interviews with 55 people working across the healthcare sector, we particularly aimed to determine if AI implementation has distinct characteristics compared to other IT applications in healthcare and identify potential bottlenecks and improvements in introducing new technologies. Our aim was to capture a broad range of perspectives relevant to the implementation of clinical AI, including those of AI developers, technology providers, clinical staff, hospital management, legal experts, and IT departments. Participants were purposively recruited based on their relevant expertise, availability, and professional connections to the participating institutions or activities related to diagnostic AI projects. Recruitment was undertaken locally by the respective partner teams using word-of-mouth sampling. The sample size was intended to ensure interdisciplinary and cross-country coverage. As healthcare frameworks and organizational structures greatly differ across European countries, the five sites in Denmark, Sweden, Germany, and Poland were selected to enable the comparison of implementation barriers and best practices across different European healthcare, organizational, and regulatory contexts.

Second, face-to-face interviews and group discussions were conducted from November 2023 to April 2024 at five CAIDX sites: with *N* = 20 in Aalborg (Denmark), *N* = 6 in Aarhus (Denmark), *N* = 9 in Lund (Sweden), *N* = 16 in Rostock (Germany), and *N* = 4 in Wrocław (Poland). Stakeholders were recruited based on their expertise, availability, and professional relationship with the institutions. They were either employed by the institution or were external collaborators on the broader topic of diagnostic AI products or related activities, such as AI developers, tool providers, clinical staff, and members of the hospital's management, legal, and IT departments. An overview of interviewers and interviewed individuals, their primary roles and additional expertise, is provided in the Supplementary Data S1. Notably, clinical AI is a highly interdisciplinary field, and many interviewees had expertise in various disciplines and contributed to the discussion from multiple perspectives.

The interviews were conducted by trained moderators (DG, CØB, THJ, ES, and KHS) at the respective sites (see Supplementary Data S1), using a shared semi-structured interview guide (see Supplementary Data S2). Each moderator held a Master's degree in health management, medical science, or a related field, and had extensive practical experience in healthcare project management (see Supplementary Data S1). The Aarhus partners (THJ and IL) initially defined the interview guide, which was then commented on and extended by the other partners before being revised and finalized prior the interviews being conducted. At the beginning of the interviews, all participants were introduced to the project and the implications of their involvement, such as the time commitment, and voice recording procedures. All interview partners gave oral consent. The Internal Review Board of the Rostock University Medical Center approved the study protocol (Registration Number: A 2023-0184). The interviews included individual and group sessions. The recordings were transcribed, anonymized, and analyzed using thematic content analysis (18, 19).

Third, we applied the following procedure to analyze and combine the interview materials and interviewees’ perspectives. The primary set of *N* = 16 interviews and group discussions conducted by the Rostock partner were used for the formal analysis. Fourth, the preliminary results were shared among the other CAIDX partners to match with their own interviews, discuss topics/aspects and their interpretation, and finally consent on the final topics, thus integrating the partners’ findings into the consented final report presented here. This procedure was necessary because the contributing sites had varying degrees of expertise in qualitative research methodology; and differences in interview language, communication culture, federated assessments, and procedural harmonization posed considerable challenges, especially given the CAIDX project timeline and allocated resources.

### 2.2 Thematic content analysis

Data was analyzed using a thematic content analysis approach (18, 19). This methodology enables a rich description of situations, actions and experiences and is suitable for analyzing many different types of texts (19). The interview recordings were transcribed verbatim by a student assistant and then imported in MAXQDA Analytics Pro 24.7.0 (VERBI GmbH) for further analysis. DG performed the first-level and second-level coding, transformed categories into themes and extracted relevant topics. A first draft of the results of topics and interviewees’ quotes was provided to all CAIDX partners for a critical review and commenting phase.

Each international partner's lead reviewed the data from the thematic content analysis. Based on the interviews conducted at all sites, the co-authors from Denmark, Poland, and Sweden reviewed and commented extensively on the representativeness, interpretation, and generalizability of the results, considering their own interview findings summarized in notes. In cases of discrepancy regarding context or interpretation, additional information was added to the final report. Thus, after the consolidation phase, the perspectives and experiences from all five interview sites were integrated into the consented final report, which is presented here.

Based on our findings from the interviews, i.e., outlined challenges and proposed potential solutions, the CAIDX partners developed a clinical AI pathway guide illustrating the individual steps required for developing and implementing AI tools in medical clinics. We co-designed this with relevant experts to ensure important aspects were covered. The clinical AI pathway guide has been published on the consortium's webpage (https://clinicalai.eu).

## 3 Results

### 3.1 Thematic analysis results

We have identified five different topics, which will be covered in the following sections: Stakeholders and teamwork, added value of AI, lessons learned, need for optimization, and features of AI. An overview of individual interview partners, their primary role, and expertise is provided in Supplementary Data S1.

#### 3.1.1 Topic 1: stakeholder and teamwork

Implementing AI in hospitals involves a broad range of internal and external stakeholders. Physicians were described as central actors throughout development, testing, procurement, and use. Their roles can vary considerably. Some act mainly as end users, expecting practical support with diagnosis, workflow or decision-making. Others participate in research and co-development with industry, while senior clinicians may contribute to managerial and economic evaluations of new tools. Physicians therefore occupy both clinical and strategic positions in the adoption process.

Other important hospital stakeholders include IT departments, procurement, legal services, data protection officers, finance departments, and management (Figure 1). Depending on the institutional structure, project management units, innovation centers, and data integration centers may also be involved. External stakeholders include commercial vendors, research partners, and, in some countries, specialized regulatory or medical device teams (Figure 1). While the exact organizational arrangements vary by hospital and country, the same broad stakeholder groups recur across settings.

**Figure 1:**
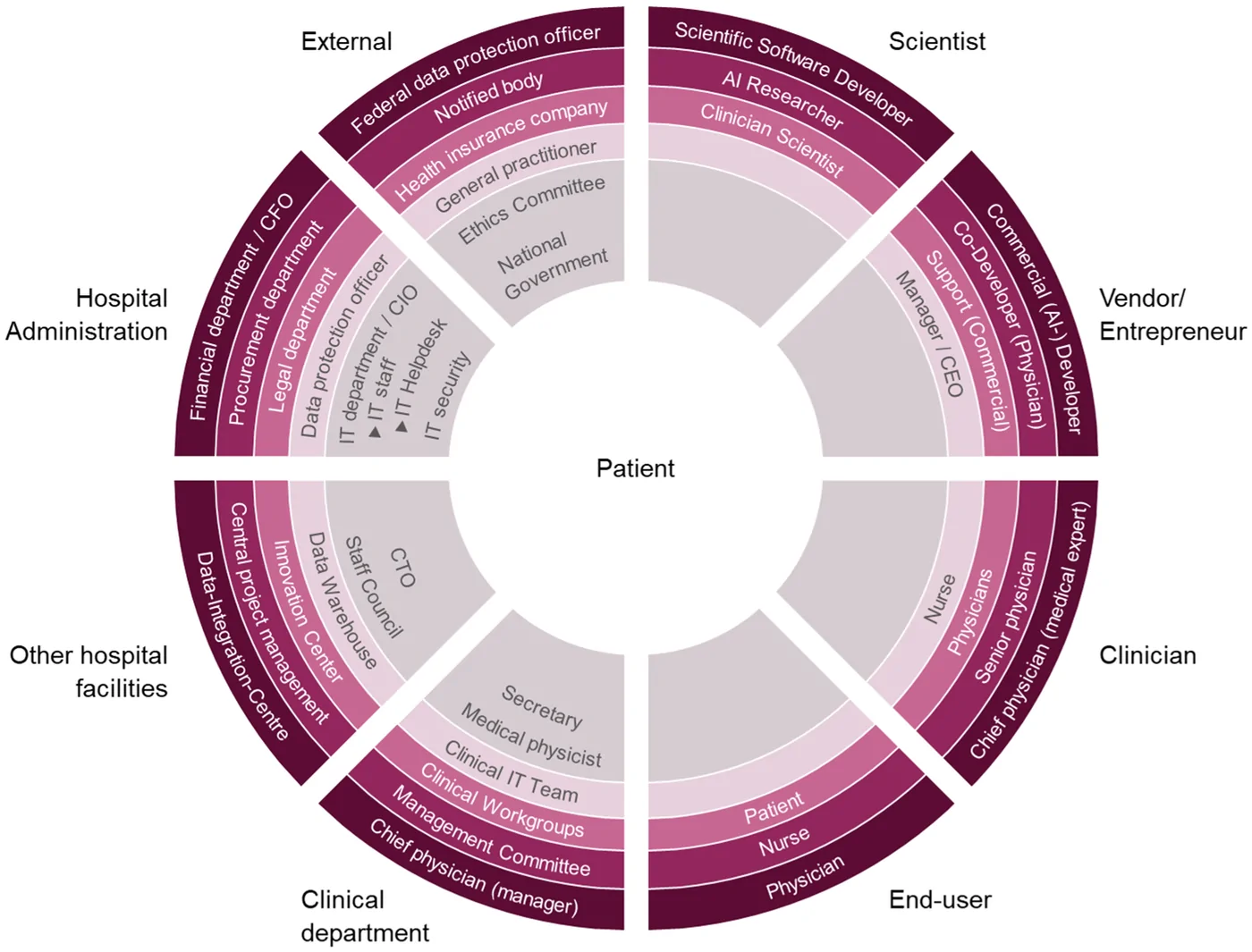
Roles and departments involved in the implementation of an AI application in hospitals. Please note that interview segments could be assigned to multiple roles. CIO, chief information officer; CFO, chief financial officer; CTO, chief technology officer; CEO, chief executive officer.

The organizational structure of hospitals varies greatly, even within a single country. However, high-level stakeholder groups are involved in the implementation process in all examined hospitals. Facilities listed under ‘Other hospital facilities’ (Figure 1) are sometimes available. There seems to be significant overlap between project management facilities, data integration centers, and innovation centers.

Collaboration patterns were heterogeneous and often poorly standardized. In many cases, chief physicians interacted with administrative and technical departments, while vendors communicated directly with intended users. The extent of collaboration with external parties depended on whether the hospital intended to purchase an existing product or engage in research and development. Procurement pathways were often described as opaque, inconsistent, and difficult to reconstruct, even by those directly involved. Consequently, different actors within the same hospital sometimes had different understandings of how implementation actually worked.

Several recurring tensions complicated teamwork. First, clinicians were often treated as scarce resources whose time should be protected. This sometimes led to their exclusion from the early stages of development and procurement. However, insufficient clinical involvement was also cited as a reason why some products failed to meet everyday needs or lacked maturity. This created a structural dilemma: hospitals wanted to avoid burdening physicians, yet inadequate physician participation could result in tools of limited practical value.

**[Managing Director of company, CaiMe01]**: “The problem is that when you ask physicians or invite them, the head physician or someone in research often shows up, because they have time to go to the event and discuss AI. But (…) you also want to talk to the people who use it every day, such as those in the emergency room.”

**[Radiologist, CaiRa02]**: “I often get the impression that there are relatively few clinicians on board with the developers and that very basic things are missing.”

Secondly, stakeholders often operated with different professional logics. Clinical users generally favored stable, reliable tools that fit established routines. In contrast, IT staff and vendors worked within environments characterized by ongoing updates, legal adjustments, and technical iteration. These different temporalities and expectations could undermine alignment between product design, technical maintenance, and clinical adoption.

**[Urologist, CaiMe07]**: “The society expects us to give a lot of time to new things, yes, to things that will improve the society. […] But still, it consumes our time. But when we invest too much time in basic research here, then we don't have the time for patients. But on the other hand, if we create a very nice tool that saves our times, we have the time for the patient.”

Thirdly, responsibilities were often fragmented. Legal, IT security, and data protection units had substantial influence over implementation, yet accountability for the overall process was not always clearly assigned. Because many AI systems require integration with hospital infrastructures or the transfer of patient data, uncertainty regarding privacy, cybersecurity, and system integrity often slowed implementation. Implementation appeared more manageable where responsibilities were clearly defined, but this was not the norm.

**[Radiologist, CaiRa02]**: “Then it's a constant exchange between the purchasing department, the IT department, the suppliers, and us. Everyone says what they still need and who needs to do what until it works.”

Fourthly, stakeholders feared investing time and effort in projects that might never reach routine use. Promising applications could fail due to high costs, poor workflow integration, unmet data requirements, technical incompatibilities, or regulatory concerns. Because evaluations often required lengthy testing and negotiation, unsuccessful attempts could consume substantial resources and reduce motivation for future initiatives. Staff turnover, particularly among junior physicians in temporary positions, further weakened continuity and institutional memory.

**[Radiologist, CaiRa02]: “**Sometimes you think that it's not worth trying out a new application, even if you really like the idea, because it's not feasible in terms of price and is wasted working time. That's a bit, perhaps not demotivating, but frustrating.”

**[Hospital CIO, CaiAd01]:** “AI-based systems in particular require familiarization, a joint learning process [user and application]. The experience of my IT staff is that physicians in particular do not undertake this training, i.e., the adaptation to their own needs, e.g., for speech recognition to their own voice.”

Overall, AI procurement and implementation were characterized by extensive interdependence but limited transparency, resulting in prolonged timelines and uncertainty. Respondents reported that implementation commonly took many months and could extend beyond a year, similar to other medical technologies, though with the expectation of increasing complexity under evolving AI regulations.

#### 3.1.2 Topic 2: added value of innovative AI tools

Respondents associated the adoption of AI with both broad innovation goals and specific expected benefits. In some cases, hospitals pursued AI because it is a forward-looking technology that signals institutional modernity. Engaging in AI was also linked to maintaining relevance, keeping pace with technological change, and participating in future-oriented medical development. Even when explicit goals were not clearly articulated at the outset, acquiring AI was usually accompanied by expectations of added value.

Three intended beneficiaries emerged across stakeholder groups: patients, clinical users, and developers or institutions seeking meaningful innovation. AI was expected to support clinical work, improve quality, save time, and ultimately contribute to better patient care. The most common aspiration was improved outcomes combined with a reduced workload. Respondents often described AI as valuable when it created benefits for multiple stakeholders simultaneously, especially when technical innovation aligned with practical utility.

Differences in emphasis reflected the initiative's starting point. When clinicians initiated adoption, the focus tended to be on medical quality, diagnostic support, patient safety, and reducing the increasing workload. When management or administrative personnel led the process, efficiency, resource use, and economic sustainability became more prominent. These perspectives were not mutually exclusive. Rather, respondents frequently framed clinical and economic value as interconnected.

Clinicians emphasized AI's potential to improve the quality and consistency of care while freeing time for direct patient contact. They considered AI especially useful in contexts of high workload, fatigue, staff shortages, or time-critical decision-making, such as emergency care and night shifts. Some respondents believed that AI has already approached or exceeded human performance in narrowly defined tasks, and they expect this trend to continue. Others highlighted the value of AI as a second reader or safety layer for diagnostic support, which could reduce oversight and improve confidence.

**[Radiologist, CaiRa02]**: “But the goal is always that it can help in a meaningful way, reduce the workload and also [improve] the quality of what we do, and ultimately benefit the patient. On one hand, that the results are better, and on the other, that it simply goes faster.”

**[Researcher, CaiRa03]**: “I would present this to the commercial director in such a way that he understands it, i.e., using statistics. How much time will this save me? How can I use it to improve patient care? And how many more patients can I possibly see or treat as a result of the time saved?”

Economic value was discussed at the levels of the individual clinician, the hospital, and the broader healthcare system. Key themes included time savings, workload reduction, and improved throughput. Stakeholders also noted that the costs of not adopting useful technology could be substantial if inefficiencies, missed diagnoses, or an increased staff burden persisted. Thus, AI was viewed not only as an expense, but also as a means of avoiding future losses.

**[Urologist, CaiMe07]**: “It's not just how much it costs to use it; it's also how much it costs you not to use it. This can be shown to the payers. We can save money this way.”

Some participants, particularly those from industry, identified prevention and long-term health maintenance as promising areas for AI. However, they also noted that current healthcare financing structures may not sufficiently reward preventive innovation. This highlights a broader tension between technological potential and reimbursement realities. Overall, the usefulness of AI was perceived based on its ability to improve care, reduce strain, and justify its costs through meaningful clinical and organizational benefits.

#### 3.1.3 Topic 3: lessons learned

Interviewees identified various barriers that complicated the adoption of AI. Some were considered difficult to change in the short term and were viewed as structural features of hospital environments. Others reflected local organizational weaknesses.

At the personal and interpersonal levels, communication across professional groups was a recurring challenge. AI implementation requires cooperation between clinicians, IT specialists, managers, legal experts, and external developers, making it highly interdisciplinary. These groups often use different terminology, hold different assumptions, and rarely engage in sustained face-to-face communication. As a result, even when actors are committed to the same overall goal, misunderstandings can arise.

**[Computer Scientist, CaiMe02]**: “I think the most important thing is trying to find the same language and taking the time to actually connect with them [users], because I think it’s at that intersection where the best ideas are born.”

Another recurring issue was the absence of personnel dedicated to introducing innovations at the departmental level. AI procurement was often driven by a motivated physician or other individual, but this person often lacked the time, technical expertise, or procedural knowledge necessary to manage the entire process. Implementation therefore depended heavily on personal commitment and informal support from cooperative staff, especially within IT. This reliance on individual initiative made projects vulnerable and inconsistent.

**[Radiologist, CaiRa02]**: “You can count yourself lucky if you have someone [in the IT department] who shares your affinity for such projects and says: ‘Yes, it’s not exactly trivial, but we can do it’.”

Hospitals were also described as highly complex, resource-constrained organizations. They face continuous legal, administrative, and technological demands while coping with workforce shortages and operational pressure. In this context, any new application creates long-term obligations for support, maintenance, training, and adaptation, in addition to upfront work. Because hospitals seek to avoid adding unsustainable burdens, the threshold for introducing new technologies is high.

**[Clinical Researcher, CaiRa03]**: “The [procurement] process is based on a high degree of division of labor.”

**[Hospital CIO, CaiAd01]**: “The federal government regularly issues new requirements, such as the use of e-prescriptions and electronic certificates of incapacity for work. Compliance with these legal requirements is mandatory; otherwise, there is a risk of high fines.”

Technical and infrastructural conditions further complicate adoption. Hospital IT systems are described as being heterogeneous, difficult to navigate, and often incompatible with external solutions. AI applications typically require access to data from multiple internal systems, yet this data is often neither well-structured nor easily retrievable. Even when a vendor has developed and tested a product extensively, its performance in a local hospital environment may differ due to data quality, system architecture, or workflow variation. These factors can lead to delays, disappointment, and low motivation among staff. However, AI implementation cannot be understood purely as a technical matter. Rather, it is shaped by institutional complexity, distributed expertise, limited resources, and dependence on local infrastructures and relationships.

#### 3.1.4 Topic 4: need for optimization

Respondents identified specific areas in which implementation processes could be improved (Table 1). A major theme was the need for greater transparency. Unclear internal procedures, inconsistent interpretations of legal requirements, and uncertainty about permissible testing and collaboration created avoidable barriers. Better-defined agreements between hospitals and companies were seen as important for reducing ambiguity and preventing later conflicts in procurement, especially concerning access to patient data, expected contributions, and mutual benefits. For example, the Danish and Swedish sites implemented hospital innovation groups that centrally manage all collaboration projects, including the administrative and regulatory work necessary for AI prototype testing.

**Table 1:** Goals of optimized procurement processes for AI applications in hospitals.

Legal framework (national level)
• Transparency and narrow scope for interpretation, especially with data protection. • Promotion of digitalization incl. interface specifications. • Penalties for lack of digital transformation.

Health care system
• Holistic evaluation of costs. • Creation of synergy effects through cluster structure of hospitals.

Hospital
• Corporate communication:
• Joint definition of the goal to be achieved with AI and explicit communication about added value. • Sharing knowledge about processes in a clearly structured rather than informal way. • A structured plan for how to proceed with implementation issues. • Organizational charts and checklists for complex processes.
• Management and Administration:
• Defined responsibilities for departments and hierarchies, without gaps and overlaps. • Genuine digitization of processes, e.g., procurement processes. • Parallel processing: possibility for collaborative document processing, without loss of information. • Digital monitoring of action management, tools for project management, document control tools. • Interlocking ticket systems of different departments. • Secondary use of routine data: hospitals as data suppliers for innovative technologies.
• IT infrastructure:
• Salaries comparable with the market to potentially reduce the shortage of skilled workers. • More powerful hardware. • Data integration: only purchase applications with defined interfaces. • Rigorous integration of all existing systems: Hospital Information System (HIS), Laboratory Information Management System (LIMS), Picture Archiving and Communication System (PACS), Radiology Information System (RIS), etc.
• Clinical departments:
• Work instructions and Standard Operating Procedures (SOPs) for the use of new applications. • Use of a communication plan. • Designation of a clinician responsible for IT, ideally a senior physician. • Regular (short) meetings, in particular with managing (senior) physicians
• Users (Clinicians):
• Better IT training during studies and further IT training for employees. • Structured introduction of new tools incl. training. • Helpdesk and IT support for users. • Involving potential real users in the development of tools and in the procurement process • Recognize co-development potential.

Vendors (companies)
• Enhance interoperability and Application Programming Interfaces (APIs). • More flexible pricing. • Cooperation agreements between companies and hospital, with clear rules and objectives.

**[Radiologist, CaiRa01]**: “Although I've been there for a few years, it's a bit of a jungle for me. So it's not really clear for many people. What are you actually allowed to test in a clinic like this?”

**[Head of the Corporate Development and Process Management, CaiAd02]**: “It is unclear to the user why some hospitals introduce certain applications and others do not, citing the GDPR [general data protection regulation within the European Union]. This should apply equally everywhere. There seem to be different interpretations of the data protection regulations. This is a hindrance.”

Digital maturity also emerged as a key issue. In less digitized hospital systems, weak data infrastructures, insufficient structuring of clinical information, missing digital signatures, and limited capacity for remote or collaborative work obstructed implementation. Even in more digitally advanced settings, challenges remained regarding data integration and standardized formats. This indicates that digitalization alone is insufficient; data quality, interoperability, and process redesign are equally important.

Several respondents emphasized the importance of clearer institutional responsibility. In some clinical departments, one physician formally oversees all IT-related matters, which facilitates evaluation and decision-making. Assigning this responsibility to experienced, stable staff was considered advantageous, though it could also put additional pressure on overburdened senior clinicians.

Respondents repeatedly described interoperability and standardized interfaces as essential. Respondents from hospitals and industry criticized the lack of robust technical interfaces and argued that stronger requirements for documented connectivity would significantly ease adoption. Overall, the proposed improvements focused less on AI-specific redesign and more on creating the organizational, legal, and technical conditions necessary for feasible and predictable implementation.

To streamline the technical implementation process for AI products, interviewees mentioned the DeepC® system (https://deepc.ai). Originally from radiology, DeepC® provides a centrally manageable IT infrastructure that implements all the necessary software and interoperability components. This allows new products to be procured from their marketplace and easily deployed onsite without repeating technical work for each product.

#### 3.1.5 Topic 5: specific features of AI

Participants identified several features that distinguish AI from other medical technologies. One such feature was uneven AI literacy. Clinicians generally reported having only a basic understanding of AI, which they often acquired informally rather than through structured professional training. Understanding levels varied markedly across individuals and countries. In more digitized healthcare settings, technological competence was more fully integrated into education and practice.

At the same time, many clinicians did not seek in-depth methodological knowledge. They expected certified tools to be reliable, yet still wanted some degree of transparency and control. This created an ambivalent user position: clinicians wanted to function mainly as users yet also wanted enough information to feel secure when results were uncertain. This indicates a demand for layered systems that allow for simple use in routine cases and deeper inspection when needed.

**[Radiologist, CaiRa01]**: “Another point is to improve the technical understanding of radiologists. Because the vast majority of our people actually have no idea whatsoever about artificial intelligence. That doesn't come with training as a radiology specialist. You have to learn it yourself. It's not rocket science, but it's beyond my capabilities.”

**[Urologist, CaiMe07]**: “Sometimes, when the technology does not answer your question or when you have doubts about the results, you should be able to press a “More Information” button. […] It would give us security.”

Perceptions of AI were generally balanced, neither uniformly enthusiastic nor skeptical. Many respondents were optimistic about AI's potential to support clinical work, particularly in standardized, high-volume areas and diagnostic assistance. AI was often viewed as an additional check that could detect findings missed by human readers. However, concerns were also raised about overreliance, opacity, misuse, cybersecurity, data protection, and possible threats to professional roles.

Bias was a particularly important concern. Respondents emphasized that AI systems may reproduce the limitations of their training data and perform unevenly across populations. They considered accuracy alone insufficient to reveal such problems, underscoring the need for broader evaluation criteria and awareness of model limitations.

**[Radiologist, CaiRa01]**: “You have to be careful not to rely on it too much.”

**[Computer scientist, CaiMe09]**: “We have to come up with safety analytics or critical thinking about what are potentials for misuse or misinterpretation. And that process should be mandatory.”

Trustworthiness was associated with transparency, explainability, certification, and oversight. Although users wanted assurance that AI tools had been properly evaluated, many were unfamiliar with how certification works in practice. Some participants questioned whether current certification procedures adequately capture the unique properties of AI systems. There was also uncertainty about who should conduct deeper external validation and monitoring.

**[Radiologist, CaiRa01]**: “The things we use in everyday clinical practice are all certified. I don't think it's our job to question their validity. That's for other people to do.”

**[Radiologist, CaiRa01]**: “Personally, I don't pay any attention to it because I work in a setting where I don't have time to check it. I assume that every icon that lands on my desktop has been checked by someone who is responsible for it.”

When comparing AI with non-AI applications, respondents cited liability, trust, development costs, and regulation as key differences. Unlike conventional software that supports isolated work steps, some AI systems can produce outputs that resemble autonomous diagnostic judgments. This raises new questions when human and machine assessments diverge. Respondents also perceived a greater potential for misuse, as well as greater ambiguity in the regulatory environment, particularly regarding certification and data governance.

### 3.2 Recommendations for future work

During the interviews with various stakeholders and further discussions among the CAIDX project members, several recommendations were collected for key issues that need to be addressed to improve the uptake of AI solutions in healthcare.

#### 3.2.1 Recommendations for healthcare providers

When introducing medical AI, we recommend early and ongoing input from clinical users. To optimize collaboration, clearly communicate goals and make revisions to align mindsets. Personal communication establishes common ground and facilitates constructive dialogue. Processes should be well-structured with defined responsibilities to avoid diffusion of responsibility. Organizational charts, standard operating procedures (SOPs), and clear deadlines may be beneficial.

Streamlining the acquisition process is highly recommended. The objective is to limit the number of each stakeholder's contacts, which greatly reduces their efforts and shortens the process. Adopt a synergistic mindset: Streamlining one position should not create more work for another department.

Since feedback from experts and key users is crucial for successful implementation, management and administration should encourage innovation and avoid wasting experts’ time on unproductive or poorly planned meetings. Clinicians need recognition for their dedication, especially during the busy acquisition process. This makes their involvement more meaningful and reduces the feeling that their time is being wasted. This encourages long-term involvement and helps hospitals with their technological transformation.

To facilitate interdisciplinary processes, hospitals can establish central innovation offices to drive progress using digital tools and mediate disputes. This approach enables hospitals to develop specific expertise and implement a communication plan that can influence future acquisitions and foster an innovation-friendly culture. The CAIDX consortium has seen positive outcomes with departments such as “central project management” or “innovation center” in hospitals in Denmark and Sweden.

Hospitals interested in using medical data to train medical AI applications must structure their data and implement Quality Management Platforms (QMPs). Data structuring involves more than just converting text. It also involves file formats (e.g., DICOM), metadata, annotation protocols, and quality control to exclude suboptimal source data (e.g., images). De-identification must be appropriate while preserving clinical context. These steps improve interoperability, quality, and usability of medical data for AI development.

#### 3.2.2 Recommendations for vendors of medical AI

The provision of specific information by the AI vendor could alleviate many reservations and promote more responsible use of the technology in question. Thus, based on the suggestions outlined by the interviewees, we have compiled a systematic list of questions and potential solutions (Table 2).

**Table 2:** Questions from potential users to developers or vendors of AI, together with suggested solutions.

Questions from potential users	Suggested solutions for vendors
What is the purpose of the tool?	Define on-label use and context of use
What is the added value compared to gold standard?	Name and (if possible) quantify the added value
How good is the algorithm?	Report performance metrics, such as accuracy, sensitivity and specificity
What data has been fed into the application?	Provide characteristics of training data, including sample size (e.g., number of persons or cells), gender distribution and ethnicity
How was it validated?	Outline the characteristics of the final testing dataset used for validation
Which features (e.g., image areas) did an AI model rely on to make a particular decision or prediction?	Use relevance maps or other explainable AI methods (e.g., feature importance analysis, partial dependence plots etc.)
What are the limitations of the tool?	Give analysis of potential bias and other limitations
What is the potential misuse?	Show risk-benefit analysis

In addition to the requests from potential users, there are also recommendations from hospital IT experts. They require relevant information on the data flow and need interfaces (Application Programming Interfaces, APIs) as well as competent support from the company. Figure 2 summarizes the ideal characteristics of AI applications, what can realistically be expected of them, and the characteristics that render them unusable.

**Figure 2:**
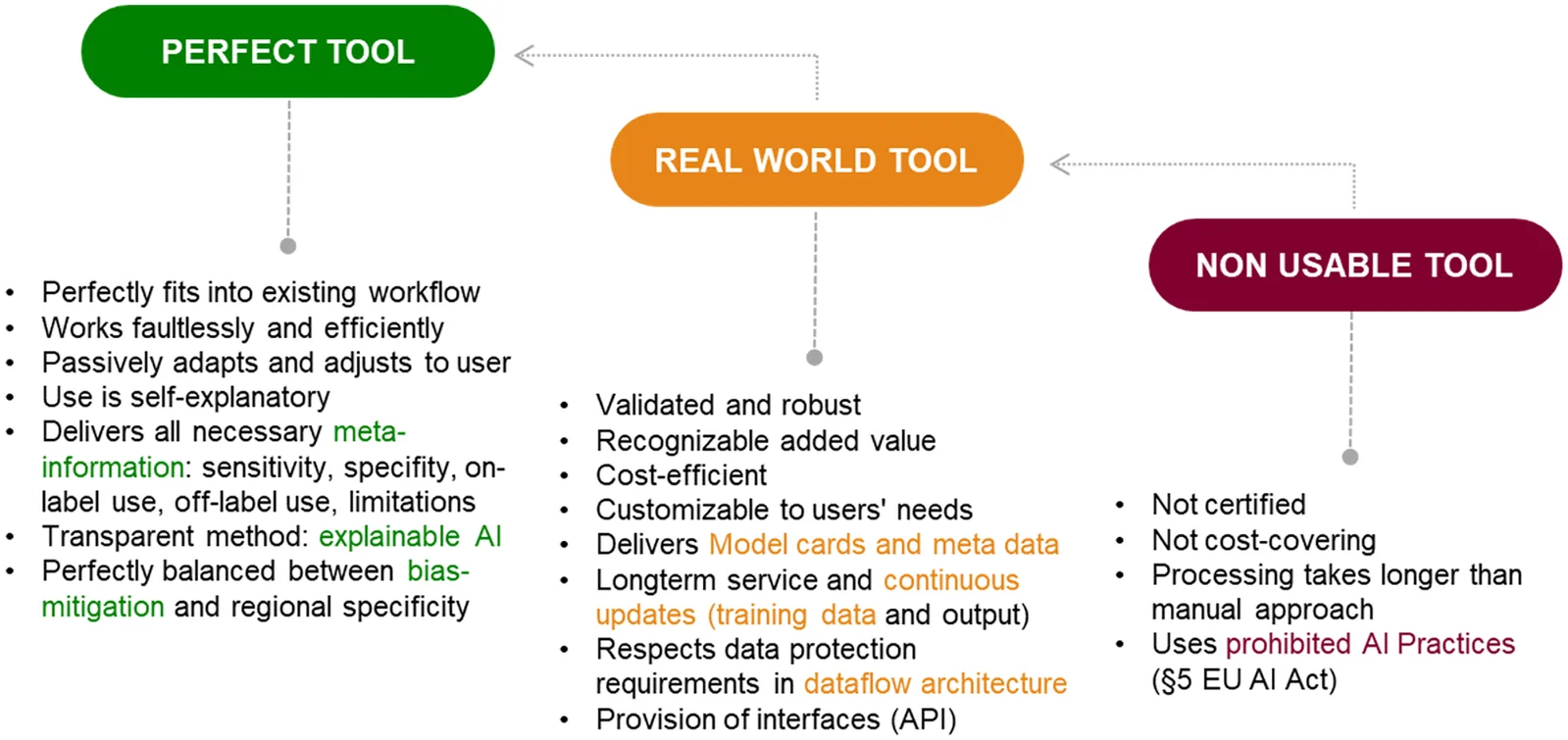
Characteristics of AI applications in medicine. Left panel: ideal characteristics, middle: realistic specifications, right: missing minimal requirements, as proposed by the study authors. Features especially relevant for AI applications are highlighted with colors.

#### 3.2.3 Recommendations for regulators

The demand for clear rules for AI solutions was initially met with the EU AI Act, which came into force 2024. Although its practical implementation poses challenges for manufacturers, institutions, and users, the EU AI Act is an important step in the medical field to ensure patient safety and ethical standards in the use of AI. Our consortium has not identified any additional proposals for regulators to improve AI applications beyond those listed in Table 1 or addressed by the EU AI Act.

## 4 Discussion

### 4.1 Key factors for the successful implementation of clinical AI

This study draws on recent, comprehensive, up-to-date survey data from four European countries, incorporating the perspectives of recognized experts from both within and outside hospital settings. We surveyed a broad range of stakeholders beyond hospitals to take a holistic view. The recommendations are for specific groups and are practical. This constructive approach sets our work apart from other publications, which carefully list obstacles and problems but offer few solutions (20).

There are many publications on “AI in medicine”, but most focus on very specific applications or aspects like trustworthiness or bias mitigation (21). Implementation and technology adoption publications often reflect the perspective of a small group of stakeholders, like physicians (22). Papers and frameworks addressing implementation problems, e.g., the Consolidated Framework for Implementation Research (CFIR) (23) or the Non-adoption, Abandonment and challenges to Scale-up, Spread and Sustainability (NASSS) Framework (24), offer important recommendations, but they are often too general and lack actionable advice (6). Our contribution complements the NASSS framework (24) by emphasizing specific topics and related recommendations in more detail. A large number of publications from Asia, particularly China, as well as the US exist. However, the findings from these regions are only partially applicable to Europe because healthcare systems differ greatly in terms of their structures and regulatory framework.

#### 4.1.1 Trust, explainability, and clinical acceptance of AI

The investigation of factors and strategies for the successful and sustainable implementation of AI-based medical applications is essential in order to leverage the full potential of innovative technologies for the benefit of patients and healthcare systems. Our interviews with various stakeholders revealed that potential users may be reluctant to adopt the technology due to concerns about its maturity, reliability, and methodology. Our surveys cannot fully explain this reluctance, but it certainly cannot be explained solely by the technology's complexity and innovative nature. Reservations about AI go far beyond this. It is possible that the different handling is attributable to the fact that AI is characterized by a mythos of the black box and inexplicability (20). It is also possible that the respondents simply had more experiences with prototypes and immature AI applications and are generalizing these poor experiences. Although hardly anyone disputes the necessity of explainable AI (XAI) in the healthcare sector, XAI is still a research field and has not yet been widely adopted (25, 26).

#### 4.1.2 Clinical validation, quality assurance, and shared responsibility

The literature shows that many applications have a validation gap (9, 27, 28), do not perform as well in the real world as they do in a laboratory environment (13, 29), and potentially have strong biases in their training data (21). Interestingly, most physicians seem to see themselves as users and do not want to have to verify an application's reliability and validity. However, current medical device regulations and certification rules are not well suited to AI applications (30), meaning that quality control must be carried out by the users today. Many physicians seem unaware of this responsibility and assume that “the hospital” or “the manufacturer” has already addressed this issue. This has created a dangerous gap in responsibility. Just adding more rules to the certification process is not a solution because developers already criticize these procedures as being too complex and expensive, especially for small businesses (31).

Recent regulations, such as the EU AI Act, now enforce a risk-based approach to high-risk AI systems used in healthcare (14). Within this framework, the responsibilities of AI providers, institutions, and clinical end users regarding safety, effectiveness, transparency, accountability and sustained human control are now more clearly defined.

#### 4.1.3 AI literacy, standardized documentation, and informed use

Since clinicians cannot rely on labels to ensure high-quality standards, they must be trained to perform their own checks. Currently, there is a lack of structured training programs, time, and relevant information from manufacturers (32). This could be achieved through mandatory, standardized documentation similar to the patient information leaflet that comes with medication. Model cards for AI applications have been proposed for several years. Model cards have been described as short documents that accompany trained machine learning models and provide benchmarked evaluations in various conditions (33). They provide structured information about a model in order to make its performance, areas of application, limitations, and ethical considerations transparent. Although model cards are not yet widely used, they are becoming increasingly important and are being actively promoted by several initiatives, such as the American “Coalition for Health AI” (34). These structured approaches to risk assessment and clear documentation are urgently needed. Otherwise, users may refuse to use AI applications out of fear of liability issues (35).

#### 4.1.4 Organizational readiness, leadership, and technical infrastructure

Our study shows that AI adoption in clinical practice must be driven by the entire organization, not just the users. A survey of executives revealed that top-level management, not the employees, should be held accountable for actions related to AI implementation (5). This should be done in consultation with future users. The survey showed that the biggest challenge in implementing AI is a lack of technical expertise. Centralized infrastructure providers such as DeepC® can serve as a blueprint for efficient, marketplace-like solutions that enable the technical deployment of AI products with minimal effort.

#### 4.1.5 Added value, sustainability, and economic evaluation

Our work suggests the need for more AI in clinical practice, though this hypothesis has not been proven. This idea is based on how quickly AI has become a part of daily life, including healthcare (1). Since healthcare is costly, the lack of skilled workers, and changing demographics in Europe are making it challenging, technical solutions are needed. But this does not mean that AI should be used uncritically. Whether an AI application is valuable must be evaluated on a case-by-case basis and in a specific context, taking into account the technical performance, an independent evaluation of generalizability, proof of clinical actionability, and evidence (10, 31). Furthermore, it remains unclear whether excellent medical AI applications are profitable in terms of patient outcomes, quality of care, or in macroeconomic and health economic factors (7).

It is clear that collecting and processing of highly structured, high-quality medical data is complex and requires resources and expertise. AI needs large amounts of data and must be developed by highly qualified engineers working with medical professionals. Other factors include integration into existing environments, ongoing support, communication with users, and user training. Significant operating costs (e.g., electricity) have not been thoroughly analyzed (10, 31). It's still unclear if AI will offer economic or health economic benefits. It is therefore necessary to assess whether AI will truly deliver economic or health economic benefits when viewed holistically.

### 4.2 Limitations

Regarding limitations of this study, we did not represent all stakeholders equally. We surveyed physicians more than therapists or nurses, even though they also use AI applications in hospitals. We primarily recruited interview participants through snowball sampling and word-of-mouth dissemination within the CAIDX partners’ professional networks. These networks primarily included employees from our institutions and external collaboration partners. This approach may have led to a slightly biased sample in which AI enthusiasts were overrepresented or social desirability influenced the interviewees’ statements. Our viewpoint is strongly influenced by the Baltic countries due to our consortium composition. Southern European countries were not represented in our surveys. Finally, the interviews did not include key aspects of the AI Act and its consequences, as the Act took effect after the interviews were conducted.

## 5 Conclusions

In conclusion, our qualitative study from the European CAIDX network demonstrates that successfully implementing AI in clinical practice requires more than just technical performance. It also necessitates organizational readiness, clear responsibility structures, meaningful clinician involvement, and trust among stakeholders. Our findings underscore the fact that AI adoption is a complex sociotechnical process influenced by local hospital contexts, workforce capabilities, and varying professional perspectives. Implementation strategies must address these contextual factors at the clinical practice, vendor, and health system levels to enable the effective integration of AI into routine care. These strategies must also be supported by targeted efforts to strengthen AI literacy, usability, and institutional commitment.

## Data Availability

The raw data supporting the conclusions of this article will be made available by the authors, without undue reservation.
