# Supplementary material for: Requirements and key factors for the successful implementation of AI in clinical practice: a qualitative study from the European CAIDX network
Source: Front Digit Health. 2026 Oct 27;8:1954586. doi: 10.3389/fdgth.2026.1954586 (PMC13749322; doi:10.3389/fdgth.2026.1954586)
Supplement: Supplementary file 1 [file Datasheet1.pdf]

Supplementary Material

1 Supplementary Data 1

Overview of interview participants and associated CAIDX project partners.

1.1 Interviews performed by Doreen Goerss (Germany)

	ID	Survey format	Main Role	Further expertise
1	**CaiRa01**	Individual interview	Radiologist	Clinician Scientist, Researcher
2	**CaiRa02**	Individual interview	Radiologist, Senior physician	
3	**CaiRa03**	Individual interview	Researcher in Radiology Department	Techno-Mathematician
4	**CaiAd01**	Focus group with Hospital Administration	Hospital Chief Information Officer	
5	**CaiAd02**	Focus group with Hospital Administration	Head of the Corporate Development and Medical Process Management	
6	**CaiAd03**	Focus group with Hospital Administration	Head of the Data Integration Center	
7	**CaMe01**	Panel discussion 1 at MEDICA	Managing Director of Company	Business IT specialist, MBA
8	**CaMe02**	Panel discussion 1 at MEDICA	Computer scientist	Focus: Intelligent Data Analytics
9	**CaMe03**	Panel discussion 1 at MEDICA	Researcher in Ethics group at Institute for Neuroscience and Medicine	Philosopher
10	**CaMe04**	Panel discussion 1 at MEDICA	Project manager at hospital	Techno-anthropologist
11	**CaMe05**	Panel discussion 2 at MEDICA	Deputy Director and Head of Research Department of Institute for Implant Technology and Biomaterials	Engineer
12	**CaMe06**	Panel discussion 2 at MEDICA	Industrial PhD, Project Manager	Techno-Anthropological consultant
13	**CaMe07**	Panel discussion 2 at MEDICA	Clinician within urology	Researcher, Co-Developer
14	**CaMe08**	Panel discussion 2 at MEDICA	Managing Director of Company	Industrial engineer
15	**CaMe09**	Panel discussion 2 at MEDICA	Computer scientist	Focus: Explainable AI
16	**CaiCo01**	Co-designing conversation	Clinician within neurology	Researcher
3	**CaiRa03**	Co-designing conversation	Researcher in Radiology Department	Techno-Mathematician
15	**CaMe09**	Co-designing conversation	Computer scientist	Focus: Explainable AI

Doreen Goerss recruited the participants and conducted the interviews, panel discussions, focus group and co-designing conversations. DG is a neurologist and researcher with focus on user-centered design of assistive technologies for cognitively impaired people.

1.2 Interviews performed by Carina Østervig Byskov and Monica Recabarren (Aalborg, Denkmark)

ID	Survey format	Main Role	Further expertise
01	Individual interview	CEO of a SME*	
02	Individual interview	CEO of a SME*	
03	Group interview 1	Physiotherapist within stroke unit	
04	Group interview 1	Data engineer	
05	Group interview 2	Clinician within oncology	
06	Group interview 2	Researcher/ data engineer within Gastroenterology and Hepatology	
07	Individual interview	Clinician within radiology	
08	Group interview 1	Clinician within neurology	
09	Individual interview	Clinician within ENT (Ear, Nose and Threat clinic)	
10	Individual interview and group interview 3	Head of Radiology	
11	Group interview 3	Head of Orthopedics	
12	Roundtable and group interview 3	IT-specialist within AI	
13	Roundtable and group interview 4	Legal officer, contract unit	
14	Roundtable and group interview 4	Legal officer, contract unit	
15	Roundtable	Procurement officer	
16	Individual interview	Data scientist within clinical AI	
17	Individual interview	Data Protection officer	
18	Individual interview	IT manager, large enterprise	
19	Individual interview	CEO of a SME*	
20	Individual interview	CEO of a SME*	
*SME = Small and Medium-sized Enterprise			

Carina Østervig Byskov and/or Monica Recabarren conducted the interviews. CØB has a Master degree in Health Management. MR has a PhD in Physiological Science. Both worked as innovation project managers at Ideklinikken, Aalborg University Hospital (Region Nordjylland).

1.3 Interviews performed by Thomas H Jensen, reviewed by Ida Lyngholm (Aarhus, Denmark)

ID	Survey format	Main Role	Further expertise
01	Individual interview	Senior Data Scientist	
02	Individual interview	Hospital Management/Mangers at clinical level	
03	Individual interview	Project Manager	
03	Individual interview	Project Manager	
05	Individual interview	Data engineer	
06	Individual interview	Clinical Specialist	

Thomas H Jensen conducted the interviews, reviewed by Ida Lyngholm. THJ has a Master degree in Business Economics. IL has a Master in Educational Science. Both work as project managers at the Innovation Center, Aarhus University Hospital (Region Midtjylland).

1.4 Interviews performed by Erik Stenberg, reviewed by Stina B Jonasson (Lund, Sweden)

	ID	Survey format	Main Role	Further expertise
43	**01**	Individual interview	Chief physician	Region Skåne
44	**02**	Individual interview	Compliance officer	Region Skåne
45	**03**	Individual interview	Chief Data & Analytics Officer	Region Skåne
46	**04**	Individual interview	CEO of a SME*	Founder of the same SME*
47	**05**	Individual interview	Chief technology officer of a SME*	Founder of the same SME*
48	**06**	Individual interview	Head of sales of a SME*	
49	**07**	Individual interview	Physician and researcher at a larger company	
50	**08**	Individual interview	Innovation specialist at a business support organization	
51	**09**	Individual interview	CEO of a SME*	
*SME = Small and Medium-sized Enterprise				

Erik Stenberg is a Senior Innovation Manager at Innovation Skåne and initiated the interviews. The interview recordings and notes were further reviewed by Stina B Jonasson. SBJ is a trained physiotherapist and post-doc researcher, and works a project manager at Skåne University Hospital, Lund (Region Skåne).

1.5 Interviews performed by Katarzyna Hodyra-Stefaniak, reviewed by Krzysztof Tupikowski (Wroclaw, Poland)

	ID	Survey format	Main Role	Further expertise
52	**01**	Individual interview	Hospital data management	Data Protection Officer
53	**02**	Individual interview	Clinician, Head of Urological Department	Head of Urological Department
54	**03**	Individual interview	Implementation IT manager	Manager of Regional Digital Center
55	**04**	Individual interview	Implementation Specials	Oncology Strategy Project Manager

Katarzyna Hodyra-Stefaniak studied European Law. She is experienced as Monitor of Clinical Trials and Quality Manager. She currently works in the Research and Development department of the Lower Silesian Oncology Pulmonology and Hematology Center, Wroclaw. Krzysztof Tupikowski is as a clinician, researcher and co-developer.

2 Supplementary Data 2

Interview Guide

Purpose of the interview

To collect views, experiences, insights, suggestions, advice regarding change management, implementation, and adoption of medical (diagnostic) tool based on AI.

The interview is a part of the European project CAIDX – Clinical Artificial Intelligence based Diagnostics with the overall purpose of minimize the barriers and increase the uptake of AI technology within healthcare.

We would like to understand if the change management, implementation, and adoption of AI is particularly difficult or different compared to other implementation of e.g., digital tool within healthcare. Based on that information and current knowledge, we will develop a handbook of steps for how to successfully integrate AI solutions in the healthcare sector by minimizing the barriers. Therefore, we would also investigate how a useful handbook or tool can be structured to increase the use of the tool by the end-users and developer of the AI-solutions.

Design

The interview is a semi structured individual interview with 5 persons within the different stakeholder groups: End-users, managers, AI-developer, and implementation specialists. Each partner country should have minimum 1 interview with one of each stakeholder. It should be recorded for transcription purposes. It is expected to take 45 minutes. The interviewee's answers will appear anonymous, and nothing they say will be written in a way that can lead back to them.

The interview should be held by two people, where one acts as the main interviewer, and the other should follow along ensuring all questions are asked. This person should also be asking clarifying questions not caught by the main interviewer.

Questions in cursive serve as elaborating questions, and should be asked if details are shallow. They also serve as examples if the stakeholder needs a push to give examples

Table of contents

Interview-guide for developers....................5
Interview-questions for the end-users (e.g., clinicians or other healthcare professionals)....................7
Interview-questions for managers....................9
Interview-questions for the implementation specialists (e.g., implementation researcher, consultants, or similar) 12

2.1 Interview-guide for developers

**Introduction of Developers**	
Presentation of the interviewer and interview	Name and role of the interviewer
	Purpose of the interview is to identify if implementation and adoption of AI solution differ from other implementation in the healthcare, and how a useful tool for them might be structured. Furthermore, how implementations are/can be handled in terms of change management.
Framework of the interview	The interview will take approx. 45 minutes
	It will be recorded for transcription purposes.
	Everything will be anonymized
	If any doubt during the interview, please ask. You can always choose not to answer specific questions, and you can always withdraw your consent regarding participation
Presentation of the interviewee	General information: Years of experience within your field and work title
	Has your role been more prominent in the planning, implementation, or evaluation phase?<br><br>*(If several, regard to this throughout the process)*<br><br>*Please have that/those role(s) in mind with the following questions*
**Theme**	**Question**
Implementation	In your experience, what are the main activities or methods used within the defined phases (planning, implementing, and evaluating)? *E.g., meetings with the department, engagement of other people, specific methods, organize new workflow etc. Discuss concrete methods and approaches ie.:*<br><br>- *How often do you have meetings? Who do you have meetings with? When and how long do you hold the meetings?*<br><br>In terms of what worked/didn't work what experiences do you have in planning/implementation/evaluation?
Preparation	How are you ensuring a successful implementation of your product? (E.g., training tool for planning, implementation and evaluating)

Expectations and recommendations	What has your role been in this/each phase? Especially, in relation to others stakeholder<br><br>What was your expectation to this/each phase? And what of you? How did it go?<br>(E.g., was there clear understanding, clear communication, clear process, clear responsibility, enough information, etc.?)
Evaluation	What is a successful implementation from your point of view?
Closing	What type of differences do you see in the implementation of AI technologies versus other types of new technology?<br><br>- *Specifically, AI regarding diagnostics*<br>- *How would you present the use of AI to your collaborators to prevent mistrust/uneasiness?*<br><br>What is your top three advice for someone planning/implementing/evaluating?<br><br>What would a relevant training module for implementation look like to increase the utilisation of the product itself?<br><br>What have we not covered within this subject that you would like to add? You can always contact us if it comes up later.
Practical sum-up	The answers will be used to create a training tool or handbook regarding change management when implementing and adopting AI. Would you like to receive the final product?<br><br>Thanks for your participation. May we contact you in case of further questions?

2.2 Interview-questions for the end-users (e.g., clinicians or other healthcare professionals)

**Introduction of End-users**	
Presentation of the interviewer and interview	Name and role of the interviewer
	Purpose of the interview is to identify if implementation and adoption of AI solution differ from other implementation in the healthcare, and how a useful tool for them might be structured. Furthermore, how implementations are/can be handled in terms of change management.
Framework of the interview	The interview will take approx. 45 minutes
	It will be recorded for transcription purposes.
	Everything will be anonymized
	If any doubt during the interview, please ask. You can always choose not to answer specific questions, and you can always withdraw your consent regarding participation
Presentation of the interviewee<br><br>This part can already take some minutes. Shouldn't we try to reduce it as much as possible?	General information: Years of experience within your field, current workplace, and work title
	Has your role been more prominent in the planning, implementation, or evaluation phase?<br><br>*(If several, regard to this throughout the process)*<br><br>*Please have that/those role(s) in mind with the following questions*
**Theme**	**Question**
Implementation	In your experience regarding implementation of a new technology, what are the main activities or methods used within the defined phases (planning, implementing, and evaluating)? *E.g., meetings with the department, engagement of other people, specific methods, organize new workflow etc. Discuss concrete methods and approaches ie.*<br><br>- *How often do you have meetings? Who do you have meetings with? When and how long do you hold the meetings?*<br><br>In terms of what worked/didn't work what experiences do you have in planning/implementation/evaluation?

Preparation	How are you ensuring a successful implementation of new AI/technology at your place of work? (*E.g., training tool for planning, implementation and evaluating*)<br><br>- What motivates you to adopt a new technology, when your department is preparing for this? (*E.g. resources, time-saving, better patient management*)<br>- What demotivates you during implementations?
Expectations and recommendations	What has your role been in this/each phase? Also, in relation to others stakeholder<br><br>What was your expectation to this/each phase? And what of you? How did it follow those expectations? (E.g., was there clear understanding, clear communication, clear process, clear responsibility, enough information, etc.?)<br><br>Regarding implementation of AI what fears, if any, have you heard from your co-workers?<br><br>- How do you/would you handle that situation?
Evaluation	What is a successful implementation in your point of view? And how do you monitor it?
Closing	What type of differences do you see in the implementation of AI technologies versus other types of new technology at your workplace?<br><br>- Specifically, AI regarding diagnostics<br>- How would you present the use of AI to your patients to prevent mistrust/uneasiness?<br><br>What is your top three advice for someone planning/implementing/evaluating?<br><br>What would a relevant training tool for planning/implementing/evaluating look like? (e.g., questions, checklist, illustrations, etc.)<br><br>What have we not covered within this subject that you would like to add? You can always contact us if it comes up later.
Practical sum-up	The answers will be used to create a training tool or handbook regarding change management when implementing and adopting AI. Would you like to receive the final product?<br><br>Thanks for your participation. May we contact you in case of further questions?

2.3 Interview-questions for managers

**Introduction of Managers**	
Presentation of the interviewer and interview	Name and role of the interviewer
	Purpose of the interview is to identify if implementation and adoption of AI solution differ from other implementation in the healthcare, and how a useful tool for them might be structured. Furthermore, how implementations are/can be handled in terms of change management.
Framework of the interview	The interview will take approx. 45 minutes
	It will be recorded for transcription purposes.
	Everything will be anonymized
	If any doubt during the interview, please ask. You can always choose not to answer specific questions, and you can always withdraw your consent regarding participation
Presentation of the interviewee<br><br>This part can already take some minutes. Shouldn't we try to reduce it as much as possible?	General information: Years of experience within your field and work title
	Has your role been more prominent in the planning, implementation, or evaluation phase? (If several, regard to throughout the process)<br><br>*Please have that/those role(s) in mind with the following questions*
**Theme**	**Question**
Implementation	In your experience regarding implementation of a new technology, what are the main activities or methods used within the defined phases (planning, implementing, and evaluating)? *E.g., meetings with the department, engagement of other people, specific methods, organize new workflow etc. Discuss concrete methods and approaches ie.*<br><br>- *How often do you have meetings? Who do you have meetings with? When and how long do you hold the meetings?*<br><br>In terms of what worked/didn't work what experiences do you have in planning/implementation/evaluation
Preparation	What differences would you expect to exist between where you implement? *(Department, hospitals, offices, regions)*

	What differences would you expect to exist between where you implement? (department, hospitals, offices, regions)<br><br>How are you ensuring a successful implementation at your place of work, where the outcome is a high adoption of the product? (E.g., training tool for planning, implementation and evaluating)
Expectations and recommendations	How do you decide which technology to implement at your department? Which criteria? And how do you communicate your decision?<br><br>What has your role been throughout an implementation? Also, in relation to other stakeholders<br><br>What was your expectation to this/each phase? And what of you? E.g., was there clear understanding, clear communication, clear process, clear responsibility, enough information, etc.?<br><br>Regarding implementation of AI what fears, if any, have you heard from your co-workers?<br><br>- How do you/would you handle that situation?
Evaluation	How would you prioritize the implementation phases in terms of: Time/ resources spent, people involved, discussion of needs and urgency, others? E.g., when did you start to have contact with the department, who's competences/ skills are needed, etc.? E.g., how long time does implementation take, who's competences/ skills are needed, etc.?<br><br>What is a successful implementation in your point of view? And how do you monitor it?
Closing	What type of differences do you see in the implementation of AI technologies versus other types of new technology?<br><br>- Specifically, AI regarding diagnostics<br><br>What is your top three advice for someone planning/implementing/evaluating? *Regarding contact, tools, etc. In general, concretise!*<br><br>Which tool do you consider relevant for planning/implementation/evaluation?<br><br>What have we not covered within this subject that you would like to add? You can always contact us if it comes up later.
Practical sum-up	The answers will be used to create a training tool or handbook regarding change management when implementing and adopting AI. Would you like to receive the final product?

	Thanks for your participation. May we contact you in case of further questions?

2.4 Interview-questions for the implementation specialists (e.g., implementation researcher, consultants, or similar)

**Introduction of Implementation Specialist**	
Presentation of the interviewer and interview	Name and role of the interviewer
	Purpose of the interview is to identify if implementation and adoption of AI solution differ from other implementation in the healthcare, and how a useful tool for them might be structured. Furthermore, how implementations are/can be handled in terms of change management.
Framework of the interview	The interview will take approx. 45 minutes
	It will be recorded for transcription purposes.
	Everything will be anonymized
	If any doubt during the interview, please ask. You can always choose not to answer specific questions, and you can always withdraw your consent regarding participation
Presentation of the interviewee<br><br>This part can already take some minutes. Shouldn't we try to reduce it as much as possible?	General information: Age, years of experience within your field, current workplace, and work title
	Has your role been more prominent in the planning, implementation, or evaluation phase? (If several, regard to throughout the process)<br><br>*Please have that/those role(s) in mind with the following questions*
**Theme**	**Question**
Implementation	In your experience regarding implementation of a new technology, what are the main activities or methods used within the defined phases (planning, implementing, and evaluating)? *Discuss concrete methods and approaches ie.*<br><br>- *How often do you have meetings? Who do you have meetings with? When and how long do you hold the meetings?*<br><br>In terms of what worked/didn't work what experiences do you have in planning/implementation/evaluation
Preparation	What differences would you expect to exist between where you implement? *(Department, hospitals, offices, regions)*

	How would you ensure a successful implementation, where the outcome is a high adoption rate of the product? *(E.g., training tool for planning, implementation and evaluating)*<br><br>- How do you secure motivation from the team
Expectations and recommendations	What has your role been throughout an implementation? Also, in relation to other stakeholders<br><br>What are your expectations to each phase? And what of you? How did it follow those expectations *(E.g., was there clear understanding, clear communication, clear process, clear responsibility, enough information, etc.?)*
Evaluation	How would you prioritize the implementation phases? (In terms of: Time/ resources spent, people involved, discussion of needs and urgency, others? E.g., when did you start to have contact with the department, who's competences/ skills are needed, etc.? E.g., how long time does implementation take, who's competences/ skills are needed, etc.)<br><br>What is a successful implementation in your point of view? And how do you monitor it?
Closing	What type of differences do you see in the implementation of AI technologies versus other types of new technology?<br><br>- Specifically, AI regarding diagnostics<br><br>What is your top three advice for someone planning/implementing/evaluating?<br><br>Which tool do you consider relevant for planning/implementation/evaluation?<br><br>What have we not covered within this subject that you would like to add? You can always contact us if it comes up later.
Practical sum-up	The answers will be used to create a training tool or handbook regarding change management when implementing and adopting AI. Would you like to receive the final product?<br><br>Thanks for your participation. May we contact you in case of further questions?
